# α-Aminophosphonates, -Phosphinates, and -Phosphine Oxides as Extraction and Precipitation Agents for Rare Earth Metals, Thorium, and Uranium: A Review

**DOI:** 10.3390/molecules27113465

**Published:** 2022-05-27

**Authors:** Esa Kukkonen, Emilia Josefiina Virtanen, Jani Olavi Moilanen

**Affiliations:** Department of Chemistry, Nanoscience Centre, University of Jyväskylä, P.O. Box 35, FI-40014 Jyväskylä, Finland; esa.p.kukkonen@jyu.fi (E.K.); emilia.j.virtanen@jyu.fi (E.J.V.)

**Keywords:** α-aminophosphonates, α-aminophosphinates, α-aminophosphine oxides, rare earth elements, actinoids, separation, recovery, extraction, precipitation

## Abstract

α-Aminophosphonates, -phosphinates, and -phosphine oxides are a group of organophosphorus compounds that were investigated as extraction agents for rare earth (RE) metals and actinoids for the first time in the 1960s. However, more systematic investigations of their extraction properties towards REs and actinoids were not started until the 2010s. Indeed, recent studies have shown that these α-amino-functionalized compounds can outperform the commercial organophosphorus extraction agents in RE separations. They have also proven to be very efficient extraction and precipitation agents for recovering Th and U from RE concentrates. These actinoids coexist with REs in some of the commercially important RE-containing minerals. The efficient separation and purification of REs is becoming more and more important every year as these elements have a pivotal role in many existing technologies. If one also considers the facile synthesis of α-amino-functionalized organophosphorus extractants and precipitation agents, it is expected that they will be increasingly utilized in the extraction chemistry of REs and actinoids in the future. This review collates α-aminophosphonates, -phosphinates, and -phosphine oxides that have been utilized in the separation chemistry of REs and actinoids, including their most relevant synthetic routes and molecular properties. Their extraction and precipitation properties towards REs and actinoids are also discussed.

## 1. Introduction

Organophosphorus compounds are one of the main commercial extractants used to separate rare earth elements (RE; lanthanoids, Sc, and Y) in solvent extraction on an industrial scale [1]. The solvent extraction is based on two immiscible liquid phases, one of which is the (acidic) aqueous phase containing REs to be separated, and the other is an organic phase including extractants. Many factors, such as the selectivity and loading capacity of extractants, number of extraction, scrubbing, and stripping cycles, and back-extraction of the extracted metal, affect the efficiency of the extraction process, but in a simplified picture, it is the coordination affinity of the extractant towards metal ions that determines the extraction degree and separation of metal ions into different fractions [2,3]. Because the coordination affinity is dictated by the molecular structure of the extractant, a plethora of different organophosphorus extractants have been developed and investigated for the separation of REs by now [1,4,5]. Apart from solvent extraction, organophosphorus compounds have also been utilized in other separation methods to recover and separate REs. Illustrative examples of such methods are fractional precipitation and solid-phase extraction [6,7,8,9].

Organophosphorus extractants are usually classified into neutral and acidic compounds, the latter of which contains at least one acidic proton. They can also be divided into four different subgroups, which are phosphates ((RO)_3_P(O)), phosphonates ((RO)_2_P(O)R’, phosphinates ((RO)P(O)R’_2_), and phosphine oxides (P(O)R’_3_), according to their functional groups (R=H, organic substituent; R’ = organic substituent) [3,10]. The basicity of organophosphorus extractants containing P=O and P-O-R bonds varies with the number of O atoms connected to the P atom; phosphine oxides are the most basic with one substituted oxygen atom, followed by phosphinates, phosphonates, and phosphates. An increase in the basicity is accompanied by an increase in the coordination strength of the extractant. Thus, phosphine oxides are usually the most efficient extractants for REs, but the separation of REs may be weaker with phosphine oxides as they may extract REs too effectively without significant separation compared to phosphinates, phosphonates, and phosphates.

**Scheme 1:**
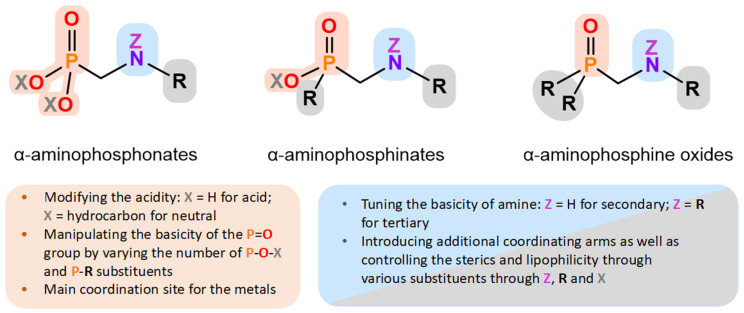
Versatile frameworks of α-amino-functionalized organophosphorus extractants and precipitation agents that can be tailored for the extraction chemistry of REs and actinoids.

The introduction of an amino group into organophosphorus compounds opens further synthetic strategies to modify their molecular structures, coordination affinity, and extraction properties [5]. For example, substituting H atoms of the amino group with new coordinating arms or long alkyl chains can increase the extractant’s affinity towards REs or its lipophilicity, respectively [5,11]. Illustrative examples of organophosphorus extractants containing the amino group are α-aminophosphonates consisting of amino and phosphonate moieties with the general formula of (RO)_2_P(O)CR’_2_NR”_2_. The R–R″ substituents can vary from H atoms to substituted hydrocarbons containing additional functional groups, making α-aminophosphonates versatile and modifiable chemical species. Replacing one of the -OR moieties of α-aminophosphonates with hydrocarbon gives α-aminophosphinates ((RO)P(O)(R’)CR”_2_NR‴_2_), whereas the replacement of two of the -OR moieties leads to α-aminophosphine oxides ((RO)_2_P(O)CR’_2_NR”_2_). As a group, these three families of α-amino-functionalized organophosphorus compounds can be classified as a subclass of organophosphorus extraction and precipitation agents that not only bear similar functional groups (P=O and amino moiety in the α position), but also have their distinct features (P-O-R vs. P-R bonds) that contribute to their complexation, extraction, and precipitation properties towards REs and actinoids (Scheme 1) [5]. Importantly, some of the α-amino-functionalized organophosphorus compounds have been proven to be better extractants for REs and actinoids than commercial extractants. 

REs play a pivotal role in several applications utilized today. Illustrative examples of such applications are ceramics [12], alloys [13], photonics [14], catalysis [15], and permanent magnets [16]. Importantly, the latter are used in electric vehicles and wind turbines, which are key players in the green technology revolution contributing to fossil-fuel-free traffic and energy production, respectively [17]. Due to the suitability of REs for a wide range of applications, it has been predicted that the demand and price of REs will significantly increase in the future. As a matter of fact, the average price of Nd, the most crucial element in Nd-based permanent magnets, has already increased from ~50 EUR/kg to a peak value of ~200 EUR/kg during the years 2018–2022 [18]. The increased demand and rise in prices of REs along with the environmental issues have considerably driven the development of separation methods, including solvent extraction, fractional precipitation and crystallization, electrolysis, and solid-phase extraction for recovering and separating REs from ores, raffinates, waste streams, and from each other during the last decade [1,19,20,21]. Despite the numerous efforts to utilize various waste streams as sources for REs, the main sources of REs are still ores, such as bastnäsite, monazite, and xenotime, as well as RE-bearing clay. The main ores of REs can also contain actinoids, such as U and Th. In particular, the content of Th can be up to 0.3 wt% and 20.0 wt% in bastnäsite and monazite, respectively, whereas U is typically found from bastnäsite (0.09 wt%) and xenotime (0.0–5.0 wt%), and sometimes from monazite, in which its content can be as high as 16 wt% [22,23]. Th has been proposed as a valuable alternative to the conventional uranium-based nuclear fuel for future nuclear reactors because it is more abundant than U, and overcomes many problems related to uranium-based nuclear fuel [24,25]. Therefore, the selective separation of actinoids from REs not only secures RE concentrates free of radioactive elements but also aims for the full valorization of RE ore by recovering every element from it.

### Scope of the Review

Taking into account all the above-mentioned, α-aminophosphonate-, α-aminophosphinate-, and α-aminophosphine oxide-based extractants and precipitation agents have strong potential to develop the extraction chemistry of REs and actinoids that are critical elements for modern society. Thus, this review aims to illustrate the essential aspects of the chemistry of α-amino-functionalized organophosphorus compounds used for recovering REs and actinoids, as well as to discuss their extraction, precipitation, and separation properties towards the aforementioned elements. Liao et al. have reviewed the subject before [5], but with a strong focus on their own work and the separation of Ce(iv) and Th(iv) from other REs. Moreover, Chistyakov et al. briefly mentioned α-aminophosphonates in their review revolving around organophosphorus extractants [4]. Compared to the previously published reviews, we will take a strong molecular approach. The review is divided into seven sections, which are: an introduction (Section 1), the history (Section 2), synthesis (Section 3), and characterization (Section 4) of α-amino-functionalized organophosphorus compounds and their complexes by IR, compositions of extracted and precipitated complexes in solution phase (Section 5), extraction and precipitation properties of α-amino-functionalized organophosphorus compounds towards REs and actinoids (Section 6), and conclusions and future perspectives (Section 7). The review covers the relevant literature on the subject published from the 1960s to March 2022, but all α-amino-functionalized organophosphorus compounds used as sorption materials in the solid-phase extraction of REs and actinoids are excluded from this review [26,27,28,29].

## 2. The Short History of α-Aminophosphonates, -Phosphinates, and -Phosphine Oxides as Extraction and Precipitation Agents

**Scheme 2:**
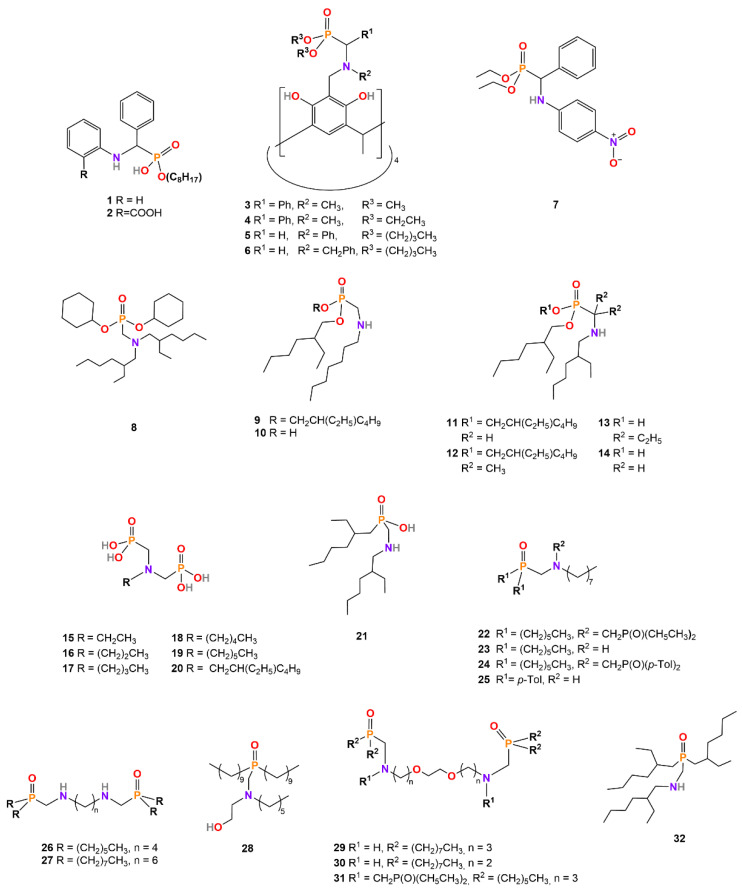
Structures of the α-aminophosphonates (**1**–**20**), -phosphinate (**21**), and -phosphine oxides (**22**–**32**) studied for RE and actinoid separation.

Scheme 2 shows all α-aminophosphonates, -phosphinates, and -phosphine oxides studied in the extraction chemistry of REs, Th, and U from the 1960s to March 2022. Among these compounds, α-aminophosphonates **1**–**20** have dominated the field since the 1960s and, in particular, during the last ten years. In sharp contrast, there is only one acidic α-aminophosphinate **21** investigated so far, and the studies performed for α-aminophosphine oxides **22**–**32** were mainly done at the beginning of the 2010s, with the exception of one study that was published in 2020.

The first extraction studies of REs and actinoids with α-aminophosphonates can be traced back to the 1960s and 1970s when Jagodic et al. investigated the extraction of REs and actinoids from the aqueous phase to the organic phase with mono-octyl ester of α-anilinobenzylphosphonic acid (**1**, MOABP) [30]. Later on, Jagodic et al. shifted their focus to the carboxylic derivative of MOABP, namely α-(2-carboxyanilino)benzylphosphonic acid (**2**, MOCABP), which was designed to extract divalent metals in addition to tri- and tetravalent metals. During the studies, Jagodic et al. not only proved the good extraction ability of MOCABP towards divalent metals from acidic solutions, but they also showed that MOCABP was a slightly better extractant for trivalent REs compared to MOABP [30,31,32,33].

After the pioneering work of Jagodic et al., interest in α-aminophosphonate-, α-aminophosphinate- and α-aminophosphine oxide-based extractants remained rather low, and it was not until the beginning of the 2000s that Fedorenko et al. published two papers focusing on calix[4]resorcinarenes, whose upper rims were functionalized with four α-aminophosphonate arms (**3**–**6**) [34,35]. The studies demonstrated that the four α-aminophosphonate arms facilitated the polydentate coordination of REs, leading to more efficient extraction of La(iii) and Lu(iii) compared to the extraction properties of O,O-diethyl[(4-nitrophenyl)aminobenzyl] phosphonate 7. The synthesized calix[4]resorcinarenes functioned as neutral extractants because the deprotonation reaction of the phenolic protons of calix[4]resorcinarenes did not occur under the extraction conditions as proven by NMR studies. Additionally, by changing the length of the alkyl chain in the phosphonate moiety and the number of counterions (sodium picrate) in the extraction process, Jagodic et al. were able to vary the metal–ligand ratio of the extracted complexes from 1:1 to 1:2. In 2009, Cherkasov et al. synthesized a family of new α-aminophosphine oxides (**22**–**28**) with one or two phosphine oxide groups and one new α-aminophosphonate (**8**) and investigated their extraction properties towards Sc(iii). They showed that the two-armed phosphine oxides were more selective compared to one-armed ones, albeit the degree of extraction of Sc(iii) was rather similar for all investigated compounds. In summary, these three studies indicated that the polydentate extractants can outperform the monodentate ones bearing similar coordinating groups, not only in selectivity but also in efficiency, by a variable margin [11].

The 2010s, particularly the late 2010s, were a renaissance in the chemistry of α-amino-functionalized organophosphorus compounds targeted for extracting REs and actinoids. In 2012, Cherkasov et al. published three different α-aminophosphine oxides **22**, **29**, and **31** and investigated their efficiency to extract Nd(iii), Sm(iii), Dy(iii), Yb(iii), and Lu(iii) from different acidic solutions (hydrochloric, nitric, or perchloric acid) to different organic phases (toluene, chloroform, or methylene chloride). Because the syntheses of **29** and **31** were challenging, their extraction studies were only carried out in perchloric acid containing Lu(iii). Cherkasov et al. found out that the extraction efficiency of the synthesized extractants strongly depended on the nature of the acidic solution [36]. The extraction efficiencies of **29** and **31** were comparable with **22** in perchloric acid. In 2013, Cherkasov et al. performed extraction studies for Sc(iii), Y(iii), La(iii), Ce(iii), Nd(iii), Sm(iii), Gd(iii), Lu(iii), and U(iv) using bisphosphorylated azapodand **30** as an extractant without and with bis(pentadecyl)phosphoric acid to investigate the synergistic effect of two extractants [37].

These two studies were followed by the discovery of Cextrant 230 (**11**), which was patented in 2017 by Liao et al. [38]. Cextrant 230 turned out to be an efficient extractant to recover +4 oxidation state ions, such as Ce(iv) and Th(iv), from the RE mixtures containing La(iii), Gd(iii), and Yb(iii) in sulfate media [39]. To explain the superior affinity of Cextrant 230 towards Ce(iv), Liao et al. compared the extraction ability between Cextrant 230 and di-(2-ethylhexyl) 2-ethylhexyl phosphonate (DEHEHP). Cextrant 230 and DEHEHP are very similar phosphonates containing one P-C, one P=O, and two P-O-C bonds, but the latter does not have an amino group. Based on the studies, they proposed that the better extraction ability of Cextrant 230 originates from its additional nitrogen atom, which can coordinate to the metal ion. However, the role of the nitrogen as a coordinating atom during the complexation has remained controversial to some extent (see below).

In the late 2010s and early 2020s, Liao et al. synthesized derivatives of Cextrant 230 by varying substituents in the amino group (**9**) [40] or methyl bridge (**12**) [41], or by converting the derivatives to acidic extractants (**10**, **13**, **14**) [42,43,44]. In the similar extraction conditions used for Cextrant 230, the derivatives **12** and **9** showed similar extraction properties to Cextrant 230 towards REs and actinoids, as the extraction efficiency of metal ions decreased in the following order Ce(iv) > Th(iv) > Sc(iii) > other RE(iii). Interestingly, among **9**, **11**, and **12**, the last one was much more selective towards Ce(iv) than Sc(iii) and Th(iv) [39,40,41,45]. Liao et al. concluded that the bigger ionic radius of Th(iv) hinders the simultaneous coordination of the P=O group and the nitrogen atom [41].

The extraction efficiency of an acidic extractant can show strong pH dependency, as was observed for **10**, **13**, and **14** [42,43,44]. These three acidic extractants were mainly developed to separate heavier lanthanoids, which has been a challenge for commercial organophosphorus extractants such as 2-ethylhexylphosphoric acid mono-2-ethylhexyl ester (HEHEHP) and di-(2-ethylhexyl)phosphoric acid (D2EHPA). Indeed, the three aforementioned α-aminophosphonate extractants performed better on the separation of adjacent heavier lanthanoids than the commercial ones. The synergistic extraction properties of **10**, **13**, and **14** were also investigated with di-(2,4,4′-trimethylpentyl) phosphinic acid (Cyanex272), D2EHPA, and HEHEHP, respectively [46,47,48]. Compared to the solvent extraction containing only one extractant, the synergistic system can have several advantages, including better extraction efficiency, selectivity, and rate, improved solubility and stability of extracted complexes, a lower tendency to emulsification, and the formation of a third layer [46,47,48,49]. The synergistic studies were carried out for **10**, **13**, and **14** because Liao et al. aimed to enhance the challenging separation of heavier lanthanoids. In all three studies, they proved that the synergistic systems outperform the extraction efficiencies of single extractants, but the results for RE separation varied.

To the best of our knowledge, only one acidic α-aminophosphinate-based extractant (**21**) has been published so far in 2022 [50]. The development of this new extractant was driven by the findings from the previous studies carried out for the α-amino-functionalized organophosphorus extractants, which showed that most of the time, the α-amino-functionalized counterparts outperform traditional commercial extractants. Liao et al. compared the extraction performance of **21** to its structural analogue di-(2-ethylhexyl)phosphinic acid (P227). Although **21** did not separate the studied heavier REs as well as P227, **21** reached the extraction equilibrium in less than 5 min, and heavy REs loaded in the organic phase with **21** were easy to strip with inorganic acids within the pH range of 0 to 2 depending on the ionic radius of the REs. Prior to this study, in 2020, Liao et al. developed α-aminophosphine oxide **32** using the same reasoning as for **21**, but they also aimed for a higher extraction performance with **32** due to the strong basicity of the P=O group. Just like Cextrant 230, **32** extracted Ce(iv) effectively from the sulfate medium, but it was also easy to strip from the organic phase [51].

α-Aminophosphonates have also been used as precipitation agents for REs and actinoids [9]. In 2021, Moilanen et al. published a study focusing on the double-armed α-aminophosphonates (**15**–**20**) with short alkyl chains to increase their water solubility. The good water solubility of the investigated compounds enabled the precipitation of actinoids and REs directly from the acidic water phase, resulting in the very good separation of Sc(iii), U(vi), and Th(iv) from REs, although the separation of the adjacent REs was minor.

It is evident from the above text that the extraction chemistry of REs and actinoids with α-amino-functionalized organophosphorus compounds that function either as extractants or precipitation agents evolved slowly at first, but during the last ten years, considerable progress has been made. In particular, the studies have shown that the extraction properties of α-amino-functionalized organophosphorus compounds can readily be changed by modifying their molecular frameworks with the well-established synthetic methods developed for the organophosphorus compounds.

## 3. Synthesis of α-Aminophosphonates, -Phosphinates, and -Phosphine Oxides

So far, three different synthetic approaches—Kabachnik–Fields, Pudovik, and Mannich—have been used to synthesize the α-aminophosphonates, α-aminophosphinates, and α-aminophosphine oxides studied in the extraction and separation chemistry of REs, Th, and U (Scheme 2 and Table 1). Among the utilized methods, the Kabachnik–Fields method has been the most used one.

**Scheme 3:**
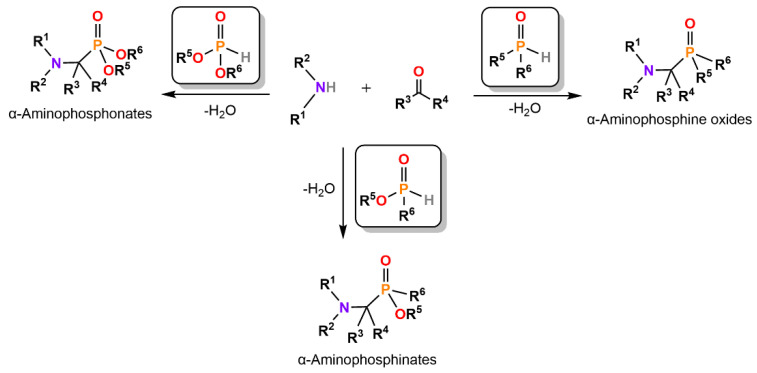
The general route for Kabachnik–Fields reaction for α-aminophosphonates, -phosphinates, and -phosphine oxides. Substituents **R^1^**–**R^6^** can be either H, alkyl, or aryl substituents.

The Kabachnik–Fields reaction includes a condensation reaction between primary or secondary amine, aldehyde or ketone, and either phosphite, phosphinate, or phosphine oxide resulting in α-aminophosphonates, -phosphinates, or -phosphine oxides, respectively (Scheme 3) [57,58]. This acid-catalyzed condensation reaction is advantageous to the synthesis of the aforementioned compounds for five reasons. (1) It is a simple one-pot reaction. (2) A variety of reagents with different substituents can be used in the reaction. (3) The basicity of the synthesized compound can be modified by varying the nature of amine and phosphorus groups; tertiary amines are more basic than secondary amines, and the number of P-O-R groups influences the basicity of the P=O group. (4) Lipophilicity and steric bulk of the compound can be altered via the substituents R^1^–R^6^. (5) More than one coordinating phosphonate, phosphinate, or phosphine oxide group can be attached to the compound by changing the stoichiometry of reagents [59].

α-Aminophosphonates **8**–**14** together with α-aminophosphinate **21** and α-aminophosphine oxides **22**–**32** were synthesized using the same procedure, by refluxing the reagents either in benzene, toluene, or acetonitrile and using *p*-toluenesulfonic acid as the acid catalyst [11,36,37,39,40,41,42,43,44,50,51]. The progress of the reaction was monitored by measuring the amount of water formed into the Dean–Stark trap. The reaction was complete when the formation of water was no longer observed. Unreacted catalytic *p*-toluenesulfonic acid was removed from the solution by reacting it with K_2_CO_3_ under reflux conditions. Finally, the solution was washed with water to separate the formed potassium tosylate and other impurities and dried with MgSO_4_, yielding oily compounds [60,61].

For 2-ethylhexyl ((2-ethylhexylamino) methyl) phosphonic acid (EEAMPA) **21** and bis(2-ethylhexyl) ((2-ethylhexylamino)methyl) phosphine oxide (DEHAPO) **32**, the phosphorous moieties were synthesized first by forming a Grignard reagent from 2-ethylhexyl bromide by mixing it with magnesium powder in THF and refluxing for 2 h, yielding (2-ethylhexyl)magnesium bromide. For compound **21**, the synthesized (2-ethylhexyl)magnesium bromide was reacted with triethylphosphite, yielding diethyl 2-ethylhexylphosphonite, which was then converted into ethyl 2-ethylhexylphosphinate by treating it with 6 M HCl [50]. In the case of **32,** (2-ethylhexyl)magnesium bromide was reacted with diethylphosphite, yielding bis(2-ethylhexyl)phosphine oxide. After the phosphorous moieties were synthesized, the reaction proceeded through the pathway described above, by refluxing the amine, phosphine, and aldehyde reagents in toluene. To obtain the hydroxyl group, the ethyl group in **21** was hydrolyzed with KOH in ethanol using KI as a catalyst [51].

Heptylaminomethyl phosphonic acid 2-ethylhexyl ester (HEHHAP) **10** was synthesized by hydrolyzing di(2-ethylhexyl)-N-heptylaminomethyl phosphonate (DEHAMP) **9** with NaOH in boiling ethanol for 6 h [44]. After removing the solvent, dissolving the sodium salt into toluene, and treating the solution with an acid, an oily product (**10**) was obtained. By using the same hydrolysis procedure, 2-ethylhexyl-3-(2-ethylhexylamino)pentan-3-yl phosphonic acid (HEHAPP) **13** and (2-ethylhexylamino)methyl phosphonic acid mono-2-ethylhexyl ester (HEHAMP) **14** were obtained from di(2-ethylhexyl) (2-((2-ethylhexyl) amino) propan-2-yl) phosphonate (DEHAPP) **12** and di(2-ethylhexyl) (2-((2-ethylhexyl)amino)methyl) phosphonate (Cextrant 230) **11**, respectively [42,43].

α-Aminobisphosphonates **15**–**20** were synthesized using water as a solvent and HCl as a catalyst instead of organic solvents and *p*-toluenesulfonic acid [9]. To obtain compounds with two phosphonic acid groups, two equivalents of phosphorous acid with respect to amine, as well as excess formaldehyde, were used in the reaction. The compounds were obtained by refluxing the reagents for 2–12 h in the acidic aqueous solution, followed by the precipitation of the products formed by adding ethanol or concentrating the reaction solution. The final products **15**–**20** were purified by recrystallizing them from hot ethanol or ethanol–water mixture.

**Scheme 4:**
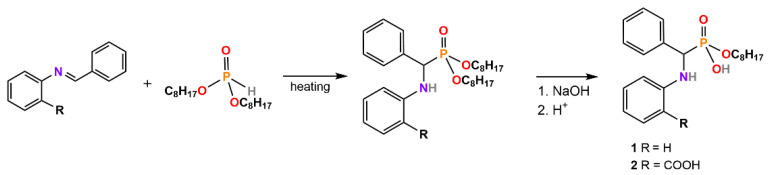
The synthetic route for α-aminophosphonates **1** and **2** through the Pudovik reaction.

α-Aminophosphonates mono-octyl α-anilinobenzylphosphonate (MOABP) **1**, mono-octyl α-(2-carboxyanilino)benzylphosphonic acid (MOCABP) **2**, and O,O-diethyl((4-nitrophenyl)aminobenzyl) phosphonate **7** were synthesized by Pudovik reaction, in which dialkylphosphite is reacted with an imine, typically under basic conditions, resulting in α-aminomethylphosphonates (Scheme 4) [62]. α-Aminophosphonates **1** and **2** were synthesized in solvent-free conditions by heating the imine and dioctylphosphite, either in a water or steam bath, for 8 h [30,63]. To obtain the mono-octyl derivatives of **1** and **2**, the dioctylphosphonate precursors were hydrolyzed by refluxing them in ethanol in the presence of NaOH for 20 h. The sodium salt of **2** precipitated out from the solution and was separated by filtration, whereas the sodium salt of **1** was obtained by removing the solvent and octanol formed in the reaction under vacuum. The sodium salts were then converted into phosphonate compounds by treating them with acid. Although the Pudovik reaction is among one of the three main approaches—Kabachnik–Fields, Mannich, and Pudovik—for synthesizing α-aminophosphonates, it has barely been utilized for the synthesis of α-aminophosphonate, -phosphinate, and -phosphine oxide extractants.

**Scheme 5:**
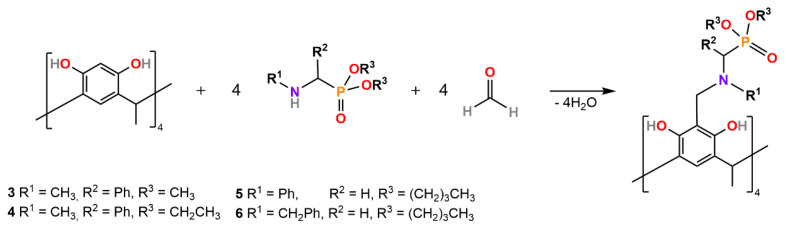
The synthesis of α-aminophosphonates **3**–**6** with the Mannich reaction.

The Mannich reaction has been utilized for the synthesis of α-aminophosphonates **3**–**6** containing a calix[4]resorcinarene moiety that provides a framework to incorporate more than one coordinating arm into a single compound (Scheme 5). Indeed, the functionalization of the resorcinarene moiety with four aminophosphonate groups enhanced the coordination ability of **3**–**6** significantly, as it was reported that the unsubstituted compound does not form complexes with La(iii) [34,35]. The Mannich reaction involves a condensation reaction between a carbonyl compound, formaldehyde, and either primary or secondary amine or ammonia under acidic conditions [64]. Typically, the aminophosphonate moieties for the ligands **3**–**6** were synthesized first with the Kabachnik–Fields reaction, by refluxing the secondary amine and phosphite reagent in the presence of an aldehyde for 2 h [34]. The obtained α-aminophosphonates were then heated with formaldehyde, while tetramethylcalix[4]resorcinarene was slowly added to the solution. After the addition, the reaction mixtures were refluxed for 12 h to yield the targeted products.

### Dimerization of the Synthesized α-Amino-Functionalized Organophosphorus Compounds

α-Aminophosphonates, -phosphinates, and -phosphine oxides that contain the terminal P=O functionality, along with N–H or P–OH functionalities, can form dimers through P=O - - H–N or P=O - - HO–P hydrogen bonds, respectively [30,53,65]. Dimerization for acidic α-aminophosphonates **1** and **2** was studied by performing molecular weight experiments in chloroform [30,53]. In solvents with a low dielectric constant, **1** likely forms the dimer through the P=O - - HO–P moieties of two molecules, resulting in an eight-membered ring structure. Additionally, it was shown that increasing the amount of **1** in the organic phase increased the formation of the dimer [32]. However, for **2**, it was observed that the compound forms dimers in concentrations between 0.001–0.01 M, but in higher concentrations, **2** starts to polymerize through the COOH and P(O)OH moieties [53]. Unfortunately, no dimerization studies for the other studied ligands containing the P=O, NH, and POH functionalities have been performed, but some of the recorded IR spectra of the investigated ligands indicate the formation of a dimer (see below).

## 4. Characterization of the Extracted Metal Complexes by IR

IR spectroscopy is a powerful and practical tool to characterize the synthesized α-amino-functionalized organophosphorus compounds and provide insight into their coordination modes with the extracted metals. The IR sample of an extracted complex can be taken directly from the organic phase onto a KBr crystal and heated with infrared light to evaporate the solvent [43,44]. This procedure results in a dry product from which the IR can be measured. So far, IR spectroscopy has been utilized to study the α-aminophosphonate and -phosphine oxide complexes of Sc(iii), Ce(iii), Ce(iv), Pr(iii), Yb(iii), Lu(iii), Th(iv), and U(vi). Distinctive absorption bands for α-aminophosphonates and -phosphine oxides arise from the stretching (*ν*) and bending (*δ*) vibrations of the N–H, P=O, P–OH, and P–O–C functionalities. When an extractant coordinates to a metal ion, the absorption bands can shift if the changes in the electron density distribution and bond lengths are strong enough to alter the dipole moment of the compound [66]. For secondary amines, the distinctive vibration band appears in the region between 3500 cm^−1^ and 3100 cm^−1^ due to the stretching of the N–H bond. Additionally, in some cases, the bending vibration of the C–N–H bonds can be identified around 1510 cm^−1^ [67,68]. The P=O functionality exhibits only stretching vibrations, which can be observed between 1320 cm^−1^ and 1140 cm^−1^ depending on the substituents attached to the phosphorus atom. More electronegative substituents can shift the absorption band to near 1400 cm^−1^, whereas substituents that can form hydrogen bonds, such as OH, shift the absorption band closer to 1100 cm^−1^. Broad absorption bands that arise from P–OH functionality due to OH stretching vibrations typically range from 2800 to 2100 cm^−1^. Absorption bands for P–OH bending vibrations can be observed around 1230 and 900 cm^−1^, although these bands are typically weak and overshadowed by P=O and P–O–C vibration bands, respectively. Strong P–O–C stretching vibrations can be found between 1088 and 920 cm^−1^. Additionally, C–H and C–C stretching and bending vibrations from the alkyl chains can be observed around 2900 cm^−1^ and throughout the fingerprint area of the IR spectrum. The shifting of these distinctive IR peaks upon a complex formation gives information about the possible coordination sites of metal ions in the studied compounds.

### 4.1. Acidic α-Aminophosphonates

Acidic α-aminophosphonates are derivatives of aminophosphonic acids, for which the most distinctive absorption bands arise from the stretching and bending vibrations of the P–OH group. Complex formation for compounds **1** and **2** was investigated with Ce(iii) and Pr(iii), for compounds **10** and **13** with Yb(iii) and Lu(iii), and for **14** with Yb(iii) [30,33,42,43,44]. Additionally, the metal complexes formed in the synergistic extractions with common extraction agents Cyanex272, D2EHPA, and HEHEHP were characterized by IR for ligands **10**, **13**, and **14**, respectively [46,47,48].

Compound **1** shows broad absorption bands for P–OH stretching vibrations at 2750–2600 cm^−1^ and 2400–2100 cm^−1^ and bending vibrations at 1750–1650 cm^−1^, whereas only stretching vibrations at 3200–2600 cm^−1^ and 2400–2100 cm^−1^ were observed for **2**. The broadness of the absorption bands most likely originates from the dimerized compounds interacting through hydrogen bonds [30]. In their corresponding Ce(iii) and Pr(iii) complexes, these absorption bands are weaker [33]. Dimeric absorption bands were also observed for **10+**Cyanex272 and both **14** and its synergistic **14+**HEHEHP system [43,47,48]. For **10**, the dimeric absorption band was observed at 1688 cm^−1^ and for Cyanex272 at 1707 cm^−1^. However, for their synergistic system, the band occurred at 1688 cm^−1^ and was not observed to disappear after the complex formation, which may indicate that **10** remains dimerized in the complex. For the sole **14** and **14+**HEHEHP synergistic system, the dimeric absorption bands were observed to occur at 1655 cm^−1^ and 1686 cm^−1^, respectively. Both absorptions were observed to disappear after the metal complex formed, indicating that the dimers break upon complex formation. For **10**, **13**, and **14**, the P–OH stretching vibrations were observed at 2438 cm^−1^, 2398 cm^−1^, and 2314 cm^−1^, respectively [42,43,44]. Additionally, an absorption band at 1643 cm^−1^ was observed for **13** and a P–OH bending band at 981 cm^−1^ for **14**. In the synergistic mixtures, the P–OH stretching vibrations were shifted to 2319 cm^−1^, 2402 cm^−1^, and 2317 cm^−1^ for **10+**Cyanex272, **13+**D2EHPA, and **14+**HEHEHP mixtures, respectively [46,47,48]. However, the absorption bands at 1643 cm^−1^ and 981 cm^−1^ remained unchanged. The P–OH stretching and bending vibrations observed for free compounds weakened or completely disappeared after the formation of the complexes, indicating that the extracted metals go through a cation exchange process with compounds **1**, **2**, **10**, **13**, and **14**, resulting in the deprotonation of the P–OH group.

Shifting of the P=O absorption bands was also observed in each complex. For **1** and **2**, absorption bands for the P=O stretching vibrations were observed at 1208 cm^−1^ and 1240 cm^−1^, respectively. In the complexes, several absorption bands were seen between 1225–1155 cm^−1^ and 1215–1160 cm^−1^ for **1** and **2**, respectively, and a new absorption band formed at 1075 cm^−1^. Compound **10** exhibits a P=O absorption band at 1216 cm^−1^ for the pure compound and at 1200 cm^−1^ for the synergistic mixture [44,48]. These absorption bands shifted to 1207 cm^−1^ and 1203 cm^−1^, respectively, upon the complex formation with Yb(iii). Interestingly, even stronger shifting of P=O absorption bands was observed for **13**, **13+**D2EHPA, **14**, and **14+**HEHEHP when they coordinated to REs. For example, the P=O absorption band for **13** was observed at 1225 cm^−1^ and for the synergistic mixture at 1231 cm^−1^, which shifted to 1175 cm^−1^ and 1176 cm^−1^, respectively, after the complex formation [42,46]. Similar shifting in the P=O absorption band occurred for **14**, although the shift was towards a higher wavenumber from 1159 cm^−1^ (pure compound) to 1204 cm^−1^ (complex) [43]. For the synergistic mixture of **14+**HEHEHP, the absorption band changed again towards a lower wavenumber, from 1206 cm^−1^ to 1145 cm^−1^ [47]. The relatively large shifts observed for the P=O absorption bands of **10**, **13**, and **14** and their synergistic systems indicated that the P=O moiety contributes to the complexation of REs. Additionally, the strong shifting of the P=O absorption bands could also originate from the breaking of the hydrogen-bonded dimer associated with the complex formation.

Unlike the P=O and P–OH absorption bands that had clearly shifted, only a small shift or no shift at all was observed for the P–O–C absorption bands of **1**, **2**, **10**, **10+**Cyanex272, **13**, **13+**D2EHPA, **14**, and **14+**HEHEHP upon complexation (Table 2) [30,33,42,43,44,46,47,48]. The strongest shift, from 1026 cm^−1^ to 1041 cm^−1^, was observed when **14** coordinated to Yb(iii), but for all other systems, the shifts were within a few wavenumbers. Thus, it was assumed that the direct contribution of the P–O–C moiety to the coordination of metal ions is negligible.

In the case of the investigated acidic α-aminophosphonates, no significant shifts or weakening in the absorption bands of the N-H functionality were observed. Therefore, it was concluded that the coordination occurs mainly through the P=O and P-O^−^ moieties of the aminophosphonic group, with no or only minor contribution from the NH group [33,42,43,44]. This is supported by the reported crystal structures of the RE complexes of the aminophosphonic acids, with varying coordination numbers from six to nine, in most of which no N-RE bond has been detected [69,70,71,72,73]. There are few crystal structures where nitrogen is coordinated to RE, but typically, these α-aminophosphonates had either multiple nitrogen atoms coordinate to RE [74,75,76] or the coordination was dictated by carboxylic acid groups due to the oxophilic nature of REs [77,78].

### 4.2. Neutral α-Aminophosphonates

In contrast to the acidic α-aminophosphonates above, α-aminophosphonates **9**, **11**, and **12** are neutral extractants. Therefore, their most characteristic absorption bands arise only from the P=O, P-O-C, and NH functionalities. IR studies were carried out for **9**, **11**, and **12** and their corresponding metal complexes (Table 2). The studied RE metal ions were Ce(iv) and Th(iv) for **9**, Ce(iv), Th(iv), Sc(iii), and U(vi) for **11**, and Ce(iv) for **12** [39,40,41,45,56]. All the solvent extraction studies were performed in sulfate medium, and therefore, absorption bands originating from SO_4_^2−^ and HSO_4_^−^ anions were also observed in the measured IR spectra.

Similar to the acidic α-aminophosphonates, a shift in the P=O stretching absorption band was observed for each α-aminophosphonate complex compared to the free extractants (Table 2). The shifts varied between 3 cm^−1^ and 113 cm^−1^ depending on the extractant and extracted metal [39,40,41,45,56]. The strongest shift, from 1239 cm^−1^ to 1126 cm^−1^, was observed for **12** upon the complex formation with Ce(iv), whereas the smallest shift (3 cm^−1^) was observed for **9** when it coordinated to Ce(iv). Based on the observed shifts in the IR spectra of **9**, **11**, and **12** upon complexation with metal ions, it was concluded that the P=O group participates in the complexation during the extraction process.

The absorption bands arising from the P–O–C moiety of **9**, **11**, and **12** were much less informative because they did not shift during the complexation, indicating no coordination affinity of the P-O-C moiety towards the investigated metal ions.

In contrast to the acidic α-aminophosphonates, where no shifting was observed for the NH absorption bands when they coordinated to metals, shifts were observed for **11** and **12** when they coordinated to Sc(iii) and Ce(iv), respectively. However, in the case of **12**, the shift in the NH bending vibration can be attributed to the sulfate ions forming a complex with **12**. In the Sc(iii) complex of **11,** the absorption band for the bending vibration of NH shifted from 1650 cm^−1^ to 1612 cm^−1^. However, the NH stretching vibration remained the same in the free extractants and all investigated complexes. The shifts in NH bending vibrations could indicate the formation of a nitrogen–metal bond, but they can also originate from the interactions of the NH group with coordinating counteranions HSO_4_^−^ and SO_4_^2−^ [39,41,45,56].

As mentioned above, the coordinating SO_4_^2−^ and HSO_4_^−^ anions also give characteristic absorption bands in the IR spectrum. For the Ce(iv) and Th(iv) complexes of **9**, the bending vibration bands from SO_4_^2−^ ions were observed to appear at 1118 cm^−1^ and 637 cm^−1^ and 1121 cm^−1^ and 640 cm^−1^, respectively [40]. The SO_4_^2−^ bending absorption bands appeared at similar regions for the Ce(iv) and Th(iv) complexes of **11**; however, only one bending absorption band could be determined for the Ce(iv) complex at 639 cm^−1^ [39]. For the Th(iv) complex of **11**, new absorption bands were observed at 1164 cm^−1^ and 641 cm^−1^, whereas the U(vi) complex of **11** exhibited one new absorption band at 640 cm^−1^ [56]. The Sc(iii) complex of **11**, in turn, exhibited one additional absorption band besides the absorption bands at 1119 cm^−1^ and 600 cm^−1^ assigned to the SO_4_^2−^ bending vibration [45]. The new absorption band appeared at 650 cm^−1^ and was assigned to the stretching vibration of the HSO_4_^−^ anion. The absorption band of HSO_4_^−^ stretching can also be observed in the Ce(iv) complex of **12** at 641 cm^−1^, in addition to the SO_4_^2−^ bending absorption bands at 1122 cm^−1^ and 588 cm^−1^ [41]. All these characteristic absorption bands proved that the sulfate ions, along with α-aminophosphonates, participate in the extraction process of REs and actinoids.

### 4.3. α-Aminophosphine Oxides and Acidic α-Aminophosphinates

Because the α-aminophosphine oxides lack both the P–OH and P–O–C functionalities, only P=O and NH absorptions are relevant for the complex formation studies, but in the case of acidic α-aminophosphinates, derivatives of the α-aminophosphinic acid, P–OH absorption bands can also be investigated. The coordination affinities of α-aminophosphinic acid **21** and α-aminophosphine oxide **32** were investigated towards Lu(iii) and Ce(iv), respectively, by IR [50,51].

Similarly to the acidic α-aminophosphonates above, **21** exhibited an absorption band for P–OH stretching at 2318 cm^−1^ that disappeared after the Lu(iii) complex was formed, indicating deprotonation of the P-OH group due to the metal coordination [50]. Shifting of the P=O group was also observed during the coordination for both **21** and **32**, as the free ligands showed a stretching absorption band at 1146 cm^−1^ and 1054 cm^−1^, which then shifted to 1162 cm^−1^ and 1040 cm^−1^ in the metal complexes, respectively [50,51]. The NH absorption bands were observed to shift from 1614 cm^−1^ to 1644 cm^−1^ for **21**, and from 3311 cm^−1^ and 1675 cm^−1^ to 3396 cm^−1^ and 1666 cm^−1^ for **32**, indicating coordination of the NH functionality to either the metals or sulfate ions. Additionally, in the IR spectra of **32**, a new absorption band formed at 1614 cm^−1^, which was assigned to arise from NH absorption.

The extraction of Lu(iii) with **21** was performed in nitrate medium, and therefore, new absorption bands from the NO_3_^−^ stretching vibrations at 1510 cm^−1^ and 1350 cm^−1^ were observed for the metal complex [50]. For **32**, which was studied in sulfate media, new absorption bands appeared at 630 cm^−1^, 584 cm^−1^, and 439 cm^−1^, which were assigned to the stretching vibrations of HSO_4_^−^ anions, whereas a new absorption band at 960 cm^−1^ was assigned to the stretching vibration of the SO_4_^2−^ [51]. These new absorption bands indicated that the NO_3_^−^ anions for **21** and HSO_4_^−^ and SO_4_^2−^ anions for **32** were included in the respective complex formations.

## 5. Composition of Extracted and Precipitated Complexes

To rationalize the extraction properties of α-amino-functionalized organophosphorus compounds at the molecular level, understanding the compositions of the metal complexes formed in the extraction process is necessary. The most popular method for this purpose has been the utilization of bilogarithmic concentration isotherms. In these graphs, the logarithm of the distribution ratio *D* of the studied metal is plotted against the logarithm of the concentration of the studied component (e.g., extractant or anion) while all other environmental conditions are kept constant. The slopes of the resulting graphs will indicate the stoichiometry of molecules involved in the complexation. However, care should be taken in interpreting the results, as the method is not without its shortcomings [79].

Another method that has been used to examine the complex formation with other organophosphorus extractants, such as carbamoylmethylphosphine oxides (CMPOs), is ^31^P NMR titration [80]. The method is based on changes in the ^31^P shift(s) when metal is titrated with a ligand or vice versa. By plotting the chemical shift of ^31^P against the concentration of RE and fitting the obtained graphs to theoretical models, the most likely metal–ligand ratios of the RE complexes are obtained.

The very first extraction study with the diaromatic α-aminophosphonate **1** did not focus on investigating the complex formation but nonetheless suggested the structures for the two uranium complexes of the extractant based on spectrophotometric and elemental analysis data. However, the data were inconsistent to some degree; the former and latter method indicated the coordination of two and four ligands to uranium, respectively [52]. In the follow-up study focusing on Eu(iii) and Tb(iii), the bilogarithmic concentration isotherms were used. The investigation revealed that both Eu(iii) and Tb(iii) form ML_3_∙HL complexes regardless of the acidic media used. The ligand HL on the second coordination sphere was found to have a substantial impact on the solubility of the complex, as pure EuL_3_, obtained via repeated ether washes of the isolated initial complex, was found to be insoluble in water and organic solvents [53]. The same extraction complex composition ML_3_∙HL for **1** and its COOH-containing analogue **2** was also obtained for Eu(iii) and Ln(iii) in several organic solvents in the later study (Table 3) [31].

The investigations on the complexation of **1** and **2** were continued using the two α-aminophosphonates in several organic solvents to extract Ce(iii) and Pr(iii) [33]. While the exact composition of the RE complexes of **1** varied, as the number of ligands on the second coordination sphere was found to be dependent on the solvent used, they always had a tri-ligand ML_3_ unit at their core as the earlier extraction studies suggested. Complex composition studies with **2** in chloroform came to the same conclusion: both Ce(iii) and Pr(iii) preferred a tri-ligand system, with the Ce(iii) complex including two extractant ligands on the second coordination sphere while the Pr(iii) complex had none (Table 3). In both cases, the phosphonic acid group of extractant is deprotonated instead of the carboxyl group that likely participates in the formation of hydrogen bonding interactions supporting the extraction process.

Almost three decades later, the focus of the extraction studies moved to macrocyclic calix[4]resorcinarenes **5** and **6**, which were functionalized with aminophosphonate groups [34]. While poor solubility prevented proper analysis of the La complex obtained with extractant **5**, compound **6** was found to form a LaL_2_Pic_3_ complex, with the three picrate anions balancing the charge of the cationic RE metal. These anions also played an important role in making the metal complex sufficiently large to be able to effectively coordinate to the cavity of macrocyclic extractant. The calix[4]resorcinarene studies were continued by using compounds **3**–**5** in the extraction of La(iii) and Lu(iii) while also comparing the results to **7** to investigate the role of the macrocyclic structure [35]. The lanthanoid–ligand ratio of the complexes was found to be dependent on the relative amount of sodium picrate used: an excess of picrate anions led to the formation of LnL_2_X_3_ complexes in most cases, whereas a lesser amount of picrate (i.e., excess of metal ions) always gave LnLX_3_ complexes (Table 3). Additionally, by comparing the extraction constants of La(iii) complexes of **3** and **7**, it was concluded that the La(iii) complex of **3** was stabilized by the macrocycle.

Structurally similar extractants **9**, **11**, and **12** were used for the separation of the tetravalent Ce(iv) and Th(iv) from trivalent RE metals. The Ce(iv) and Th(iv) complexes of **9** were found to have the structures of Ce(SO_4_)_2_ ∙ 2L and Th(HSO_4_)_2_SO_4_ ∙ L, respectively, both containing sulfate anions from the acidic medium [40]. Unsurprisingly, the extracted complexes of **11**—Ce(HSO_4_)_2_SO_4_ ∙ 2L and Th(HSO_4_)_2_SO_4_ ∙ L—were similar, with their only difference from **9** being the anions included in the Ce(iv) complex [39]. Further studies with **11** revealed that the extracted complexes of Sc(iii) and U(vi)O_2_ also contain two ligands, Sc(HSO_4_)SO_4_ ∙ 2L and UO_2_SO_4_ ∙ 2L, respectively, while the number of HSO_4_^−^ ions decreased due to the lower charge of the extracted cations [45,56]. The Ce(iv) complex of **12** was also found to have the same Ce(HSO_4_)_2_SO_4_ ∙ 2L composition as the complex of **11**, while the Th(iv) complex was not investigated [41].

Studies on congeneric monoacidic α-aminophosphonates **10**, **13**, and **14**, in turn, have concentrated on the extraction of trivalent lanthanoids. The complex formation of acidic α-aminophosphonate **10** was investigated with Yb(iii) and Lu(iii), and the RE complexes of the metals were found to have the composition of MClH_2_L_4_ [44]. The N-(2-ethylhexyl) congener **14** of **10** was found to form complexes with the same MClH_2_L_4_ composition with the trivalent Yb(iii), Lu(iii), and Tm(iii) [43]. In both cases, two dimerized extractants were partially deprotonated before coordinating to the extracted metal. In contrast, compound **13** with di-ethylated α-carbon was found to form a simple ML_3_ complex with the trivalent La(iii), Gd(iii), Y(iii), and Lu(iii) [42]. In this case, the deprotonation of the extractant was complete and broke apart the dimerization of **13**. Based on the results obtained with the aforementioned REs, all three studies generalized the observed compositions to concern all trivalent RE complexes of **10**, **13**, and **14**.

An interesting addition to the complex composition studies has been the research on synergistic extraction, where α-aminophosphonates are paired with another organophosphorus extractant. The Lu(iii) complex of **13**+D2EHPA was found to have the structure of LuCl_2_H_4_A_3_B_2_, where A depicts the amount of α-aminophosphonate and B the amount of D2EHPA [46]. The composition had the same amount of **13** as the ML_3_ complex of the pure α-aminophosphonate extractant, while also including two D2EHPA units, bringing the total number of extractants from three to five. Furthermore, only one of the three α-aminophosphonates is deprotonated in the synergistic extraction process while the other two, as well as the two D2EHPA units, stay in a neutral dimerized form. A similar trend was observed with the **14**+HEHEHP pairing, as the synergistic system complex MA_2_B_4_ requires two units of **14** and four HEHEHPs to extract a single RE cation, whereas the RE complex of pure **14**, MH_2_ClL_4_, only included four extractants in total. Both extractants of the synergistic system remain in a singly deprotonated dimer form [47]. In contrast, the **10**+Cyanex272 complex MH_2_Cl_2_A_2_B contained one neutral dimer of **10** and one deprotonated Cyanex272, which means that the synergistic system leads to a lower total amount of extractant ligands when compared with the MClH_2_L_4_ complex of pure **10** (Table 3) [48].

A study on α-aminobis(phosphonates) determined the compositions of the complexes via ^31^P NMR titrations in D_2_O [9]. This method was successfully employed for **15** with Y(iii), La(iii), and Lu(iii), and the results revealed the complex compositions of YL_3_, LaL_2_(NO_3_), and LuL(NO_3_)_2_, respectively. In each case, the extractant was in a zwitterionic form and coordinated in a bidentate manner to the extracted metal cation while NO_3_^−^ ions and/or H_2_O most likely complemented the coordination sphere of the RE. In addition, each phosphonate group was only singly deprotonated due to the pH range of the experiments. Further attempts at determining the complexes for Sc(iii) and Th(iv) were unsuccessful due to heavy precipitation of the formed complexes at low pH values.

While the research towards new α-aminophosphonates seems ever-expanding, the RE extraction properties of α-aminophosphinates remain largely uncharted. The sole reported study so far used acidic α-aminophosphinate reagent **21** for the extraction of trivalent REs from nitric acid media [50]. The complex formation was studied for La(iii), Nd(iii), Gd(iii), and Lu(iii), and all their complexes were found to have the same MHL_3_NO_3_ composition, consisting of one individual deprotonated ligand and one singly deprotonated dimer for every RE cation.

The extraction studies were expanded to α-aminophosphine oxides when compounds **22**–**28** were investigated for the extraction of Sc(iii) and other selected RE metals. The composition of the Sc(iii) complex of **22** in toluene was found to be ScL_2_X_3_, with X denoting acidic anions included to balance out the charge of the metal [11]. Attempts to use the bilogarithmic plots to investigate the Sm(iii) complex of **22**, as well as the complexes formed by **23**, were unsuccessful, as the former resulted in a nonlinear graph and the latter to ambiguous conclusions. The other synthesized extractants were not researched further [11,36]. Phosphine oxide **32**, in turn, was investigated for the extraction of Ce(iv). Unlike the Ce(HSO_4_)_2_SO_4_ ∙ 2L complexes of its α-aminophosphonate congeners **11** and **9**, the composition of **32** was found to be Ce(HSO_4_)_2_SO_4_ ∙ L, including only a single unit of the extractant [51].

In general, the studies have shown that the (mono)acidic α-aminophosphonate and α-aminophosphinate extractants favor three to four coordinated ligands around each metal cation, form complexes through deprotonation of the acid or its dimer and act as the counterions for the cationic metal centers (Table 3). The neutral di-alkoxy α-aminophosphonates and α-aminophosphine oxides, on the other hand, are more likely to stay in the range of one to two ligands per metal. Moreover, the anions of the acidic solution are often involved in the extraction process, as the extracted complexes transferred to an organic phase must be charge-neutral. This, in turn, means that the complex compositions in different acidic media are inherently varied, and the importance of the counter anion is further underlined by the findings from the extraction experiments with sodium picrate and **3**–**5**. The studies have also confirmed that the solvent of the organic phase can have an impact on the composition as well, despite not being a part of the complex itself. In short, the complex formation can be described as a complicated process with multiple experimental factors affecting the outcome of the extraction.

## 6. Extraction Ability of α-Aminophosphonates, -Phosphinates, and -Phosphine Oxides towards REs and Actinoids

To compare the recovery and separation properties of extractants and precipitation agents, several parameters have been developed to quantify their performance. The key parameters are the distribution ratio *D*, separation factor *SF,* and synergistic enhancement coefficient *R*, the last of which only applies to synergistic systems containing two extractants [81].

The distribution ratio describes the extraction ability of a compound towards certain elements. In solvent extraction, it can be determined as the concentration of extracted metal in the organic phase [*M*]*_org_* divided by the concentration of the unextracted metal remaining in the aqueous phase [*M*]*_aq_*. In the case of precipitation processes, the organic phase concentration is replaced by the amount of precipitated metal [*M*]*_p_* and calculated as the difference between initial and final concentrations of the aqueous phase, as shown by Equation (1):

(1) $$D=\frac{{\left[M\right]}_{org}}{{\left[M\right]}_{aq}}\;\;or\;D=\frac{{\left[M\right]}_{p}}{{\left[M\right]}_{aq}}=\frac{{\left[M\right]}_{init}-{\left[M\right]}_{aq}}{{\left[M\right]}_{aq}}.$$

High values of distribution ratios indicate a strong transfer of metal ions from the water phase to the organic phase or strong precipitation, whereas values close to zero are a sign of poor transfer of metal ions [81].

The separation factor is calculated with Equation (2) as the quotient of the distribution ratios *D* of the two metals *A* and *B*:

(2) $$\;SF=\frac{D_{A}}{D_{B}}.$$

The parameter describes the ability of the extractant or precipitation agent to separate two metals from each other. Separation factor values close to unity are a sign of poor separation, while significantly higher or lower values indicate that the extractant or precipitation agent can be used to efficiently separate the two metals in question [81].

The second important parameter derived from the distribution ratios is the synergistic enhancement coefficient, which aims to quantify the potential improvement of a system using two extractants simultaneously [82]. This is done by comparing the extraction performance (i.e., distribution ratio) of the combinatory system *D_AB_* to the sum of the distribution ratios of the individual components (*D_A_* + *D_B_*) according to Equation (3):

(3) $$R=\frac{D_{AB}}{D_{A}+D_{B}}.$$

Consequently, enhancement coefficient values over 1 indicate a positive synergistic effect, whereas the opposite is a sign of negative competition between the two extractants. It should be noted that the values of *D*, *SF*, and *R* are dependent on several experimental conditions, such as temperature, pH, and concentration, all of which can affect the behavior of both the metal and the extractant itself.

In addition to the distribution ratio *D*, separation factor *SF*, and synergistic enhancement coefficient *R*, another important factor measuring the performance of extractants is their loading capacity. This parameter is, simply, the maximum amount of metal that can be extracted under certain experimental conditions, and it is commonly reported in g/L or mol/L. It is therefore essential to pay close attention to the reported concentration of the extractant to determine whether the loading capacity values are directly comparable or not.

### 6.1. α-Aminophosphonates

Octyl α-anilinobenzylphosphonic acid **1** and its ethyl analogue were the first α-aminophosphonates that were investigated for the solvent extraction of REs and actinoids. While the ethyl analogue was too water-soluble for the extraction of metals, **1** was found to be a very good extracting agent for binary and ternary systems containing radioactive nuclei. Studies on U extraction in ligroin found that U(iv) was extracted quantitatively only between sulfuric acid concentrations of 2 M and 4 M, whereas U(vi) could be extracted with a broader range of 0.5–9 M acidity [52]. A follow-up study showed that **1** was able to separate U(vi) selectively from Eu(iii) and Tb(iii) when ligroin was used as an organic solvent, and the molarity of the aqueous phase was higher than 0.5 M [53]. According to the authors, the determined *SF_U/Eu_* and *SF_U/Tb_* were ~26,000 (Table 4). The full separation between Sr(ii) (the source of the radioisotope of ^88^Y) and Y(iii), as well as between ^131^Ba(ii) and ^140^La(iii), was also obtained in petroleum ether, keeping the hydrochloric acid molarity between 0.01 M and 0.1 M [54,55].

The research on the carboxylic derivative (**2**) of **1** not only revealed that it is a better extractant for Eu(iii) and Ln(iii), but it was also more selective towards divalent transition metals than trivalent REs compared to **1** [30,31]. However, the solubility of **1** was much better in different organic solvents compared to **2**, which was only well-soluble in CHCl_3_. Indeed, the studies in various organic solvents revealed distinguishable changes in the Eu(iii) extraction behavior of **1**, with increasing HCl concentration of the aqueous phase. The best extraction ability was maintained with petroleum ether and cyclohexane, with the increasing acid concentration compared to CCl_4_, benzene, and CHCl_3_ [32]. Both acidic α-aminophosphonates were also utilized to extract Ce(iii) and Pr(iii) from hydrochloric acid medium, but the differences in their extraction behavior were too small to allow efficient separation of the two metals from each other [33].

A study focusing on the extraction of La(iii) with two α-aminophosphonates functionalized macrocyclic calix[4]resorcinarenes **5** and **6** reported that La(iii) does not coordinate to nonfunctionalized calix[4]resorcinarenes, nor does it form complexes without the suitably sized lipophilic picrate counterions that fill the cavity of the calix[4]resorcinarene, as mentioned above [34]. The studies were continued with the extraction of La(iii) and Lu(iii) with compounds **3**–**5**, and the influence of the relative amount of picrate anions on the extraction properties of **3**–**5** was also investigated [35]. Interestingly, in the presence of the excess of sodium picrate, the extraction efficiency of **3** towards La(iii) was found to be higher than the extraction efficiency of **4** and **5** due to the change in the metal–ligand ratio in the complex formation from 1:1 (**3**) to 1:2 (**4** and **5**). Contrary to La(iii), **5** was the most efficient extractant for Lu(iii). Importantly, all calix[4]resorcinarene–aminophosphonates were more efficient extractants than **7**, indicating the strength of multiple coordinating arms in the extraction process.

Several studies on α-aminophosphonates have concentrated on the extraction of Ce(iv) and Th(iv) from sulfuric acid leach of the bastnäsite ore using heptane as a diluent. Compound **9** effectively separated the aforementioned tetravalent metals and Sc(iii) from the rest of the studied trivalent REs. The extraction of Th(iv) and Sc(iii) was found to decrease sharply with increasing acidity, while the extraction of Ce(iv) remained practically complete in the sulfuric acid concentration of <4 M [40]. Comparable results were obtained for the structurally similar α-aminophosphonate **11**, as Ce(iv) and Th(iv) were efficiently separated while the extraction of Th(iv) was more prone to changes in acid concentration. The extractant was successfully used to obtain RE products of high purity with high yields in a pilot test. It was subsequently patented and named as Cextrant 230 [39]. The extractant **12** performed similarly to **9** and **11** in the extraction studies because the most efficient metal separation occurred when the sulfuric acid concentration did not exceed 1 M [41]. However, a notable exception to the other two extractants was the low extractability of Th(iv) with **12**, which enabled the efficient separation of Ce(iv) and Th(iv) (SF_Ce/Th_ = 754.2, Table 4). The increased selectivity was assigned to the steric effects arising from the larger ionic radius of Th(iv), hindering its effective coordination to **12**.

Compound **11** was further studied for the extraction of Sc(iii) and U(vi). The extraction of Sc(iii) from red mud was investigated with various acids, and the results showed that dilute sulfuric acid was by far the most efficient medium. Unfortunately, **11** was also found to extract significant amounts of other REs, as well as Ti(iv) and Fe(iii), all of which are prevalent in red mud, but after a series of post-extraction treatment procedures, a purity of ~94% was achieved for the Sc_2_O_3_ product [45]. The results from the extraction studies of U(vi) and Th(iv) suggested that the separation of two actinoids from RE metals, Fe(iii) and Al(iii), is effective throughout the studied pH range of 0.22–3.23. U(vi) was best extracted and separated from REs at pH 0.22 with high *SF_U/R_*_E_ > 1000. Moreover, U(vi) had a higher loading capacity (6.16 g/L vs. 4.08 g/L for 5% extractant in heptane) than Th(iv) (Table 5) [56].

The extraction and separation of trivalent REs from each other have also been investigated with monoacidic α-aminophosphonate reagents **10**, **13**, and **14**. The extraction efficiency of **10** and **13** towards Y(iii), La(iii), Gd(iii), Ho(iii), Er(iii), Tm(iii), Yb(iii), and Lu(iii) decreased with increasing acid concentration [42,44]. A similar trend was observed for **14** when the extracted metals were Sc(iii), Y(iii), Ho(iii), Er(iii), Tm(iii), Yb(iii), and Lu(iii), while the extractabilities of La(iii) and Gd(iii) were not strongly affected by the concentration of acid [43]. The best results were obtained with extractant **13**, which has some of the highest reported separation factors among all the studied α-amino-functionalized organophosphorus extractants listed in Table 6. In general, the investigated α-aminophosphonate compounds have shown a better ability to separate adjacent heavy REs from each other than the commercially used extractants D2EHPA and HEHEHP [84].

While the majority of previous studies carried out for α-aminophosphonates have focused on solvent extraction, these compounds also work as precipitation agents. A series of α-aminobis(phosphonates) **15**–**20**, with variable hydrocarbon chain lengths, was studied for the recovery of REs, Th(iv), and U(vi) by direct precipitation of the formed complexes from the nitric acid solution [9]. α-Aminobis(phosphonates) **18**–**20**, with a longer hydrocarbon chain, separated Sc(iii) and both actinoids from the rest of the investigated REs well in the pH range of 1–2. In this pH range, Sc(iii), Th(iv), and U(vi) completely precipitated out from the nitric acid solution, while REs remained in the solution (Table 4). While similar trends in selectivity were observed for **15**–**17**, their precipitation percentages did not surpass 60% at pH < 2, where the precipitation of other trivalent REs stayed under 10%.

The synergistic extraction of REs by using binary mixtures of an acidic α-aminophosphonate and another acidic organophosphorus extractant has been studied as a potential way to improve either the selectivity of the system or the extractability of the metals of interest. For the **13**+D2EHPA system, the separation factors for studied REs were found to be lower than with pure **13**, but it still outperformed the separation efficiency of pure D2EHPA for heavier lanthanoids (Table 6) [46]. When comparing the separation factors of pure **14** and its synergistic system with HEHEHP under similar experimental conditions, the latter does not seem to bring a major improvement in performance over the former, as indicated by minor changes (less than ±0.4) in the determined separation factors (Table 6). However, the loading capacity of the synergistic system is almost doubled for Lu(iii) and Yb(iii) compared to pure **14** (Table 5), although it does not reach the Yb(iii) capacity of pure HEHEHP (32.92 g/L) [47]. The separation factors determined for heavy lanthanoid separation with the **10**+Cyanex272 system, on the other hand, showed general improvement over the separation factors obtained for pure **10** and Cyanex272. A closer inspection of data also reveals that out of these three synergistic systems studied, the binary mixture consisting of **10**+Cyanex272 performed the best in heavy RE separation [48].

The highest synergistic enhancement factors reported in the aforementioned α-aminophosphonate studies are listed in Table 7. It should be noted that the best *R* values describe the enhancement obtained compared to the individual performance of two extractants, so it does not directly correlate to the best extraction capability of the system. Consequently, while the highest *R* values were obtained with α-aminophosphonate molar fractions of 0.5–0.6, the highest distribution ratios *D* of the two-component systems of **14** and **10** were found around molar fractions 0.3–0.4. This observation is consistent with the determined 1:2 extractant ratio of the metal complex of the former system but opposite to **10**+Cyanex272′s 2:1 ratio [47,48]. Intriguingly, the maximum *D* of the **13**+D2EHPA system was found at 0.8 and, while not in perfect agreement with the 3:2 complex composition, both ratios indicate that the α-aminophosphonate component played the more important role in the overall performance of the extraction process [46].

### 6.2. α-Aminophosphinates

Contrary to the better-explored α-aminophosphonates, only one RE extraction study has been reported for α-aminophosphinates so far [50]. The monoacidic α-aminophosphinate **21** extracted REs from nitrate medium similarly to monoacidic phosphonates **10**, **13**, and **14** because the extractabilities of REs increased with increasing pH. Only the light REs—La(iii), Ce(iii), Pr(iii), Nd(iii)—could not reach complete extraction, even at pH 4 or higher. Based on the determined separation factors for the adjacent heavy REs, **21** separated them better than the typical commercial extractants D2EHPA and HEHEHP, but it did not outperform its commercial phosphinic acid analogue P227 (Table 6). However, **21** reached the extraction equilibrium much faster, and its Lu(iii) loading capacity of 0.45 mM was 1.5 times higher than P227′s 0.32 mM under the same experimental conditions.

### 6.3. α-Aminophosphine Oxides

The research on α-aminophosphine oxides started with investigations on the possibility of using the compounds as extractants for Sc(iii). A series of compounds **22**–**28**, including also one α-aminophosphonate **8**, was synthesized, and while most of them showed some capability for Sc(iii) extraction, only the two best-performing reagents **22** and **23** were investigated further [11]. Both compounds were able to separate Sc(iii) from a variety of di- and trivalent metal ions; in particular, **22** was effective in 0.3 M nitric acid medium when toluene was used as a diluent. The selectivity of extractants towards Sc(iii) was further confirmed in a follow-up study where the extraction of several trivalent lanthanoids was investigated as well [36]. The highest extraction degree (~80%) of REs was obtained from perchloric acid, surpassing the extraction degree (~30%) of two other acids, hydrochloric and nitric, by 50 percentage points when the acid concentration varied from 0.25 to 0.5 M. The extractability of the investigated REs followed the decreasing ionic radii of REs; Nd(iii) was extracted the best, followed by Sm(iii), Dy(iii), Yb(iii), and Lu(iii).

The synthesized α-aminophosphine oxide–azapodands **29** and **31** showed extraction properties towards Lu(iii) similar to **22**, but their more difficult synthetic procedure contradicted their usability in large-scale solvent extraction [36]. A follow-up study with a slightly smaller azapodand **30** in toluene showed that U(vi) and RE(iii) ions, except Y(iii), are extracted practically quantitatively from a perchloric acid solution at a pH of 4.7, whereas bis(pentadecyl)phosphoric acid extracted U(vi) and Lu(iii) from hydrochloric acid more selectively compared to other studied REs at a low pH regime [37]. Synergistic studies in hydrochloric acid showed that, by combining **30** and bis(pentadecyl)phosphoric acid in a 1:2 ratio, the selectivity towards U(vi), Y(iii), and Lu(iii) can be increased at low (2.9) and high pH (5.0–5.5) regimes, while other RE ions extracted poorly (La(iii), Ce(iii), and Nd(iii)) or moderately (Gd(iii) and Sm(iii)) from the aqueous phase.

Like its α-aminophosphonate congener **11**, α-aminophosphine oxide **32** was studied for the extraction of Ce(iv) from bastnäsite ore [51]. The extractant was found to have high selectivity towards Ce(iv), with *SF* exceeding 100 for all studied metal pairings, allowing the effective separation of Ce(iv) from Th(iv) and several REs. Although α-aminophosphonate **12** achieved better Ce(iv)/Th(iv) separation in dilute sulfuric acid, **32** was able to keep the separation relatively high despite increasing acidity. Compound **32** also outperformed **12** in the Ce(iv)/RE(iii) separation in virtually all studied acid concentrations. However, the Ce(iv) loading capacity of 16.66 g/L was notably lower for **32** than for the three α-aminophosphonate extractants **9**, **11**, and **12**, as all of them reached the loading capacities of 30 g/L or higher (Table 5).

To summarize Section 6, the various α-amino-functionalized organophosphorus extractants have generally demonstrated good selectivity towards REs and actinoids, particularly U(vi), Th(iv), Ce(iv), and Sc(iii). The studied systems have proven to outperform commercial extractants such as D2EHPA and HEHEHP in several aspects, with the improved separation of adjacent heavy REs as one of the most important highlights. While the experimental extraction data of α-aminophosphinates and phosphine oxides—*SFs* in particular—are still more scarce compared to the more studied α-aminophosphonates, the results so far indicate similar performance levels and encourage further studies on their extraction chemistry. The utilization of α-amino-functionalized organophosphorus compounds as part of synergistic extraction systems is another rather unexplored area with few but promising results.

## 7. Conclusions and Future Perspectives

The interest in the α-amino-functionalized organophosphorus-based extractants and precipitation agents has grown rapidly during the last ten years after the slow start initiated in the 1960s [5]. At the heart of this process have been synthetic methods such as Kabachnik–Fields and Pudovik, developed more than half a century ago, that allow the facile synthesis of a myriad of different α-amino-functionalized organophosphorus compounds [57,58,64]. Despite the progress in synthetic chemistry, their utilization in the extraction chemistry of REs and actinoids has just scratched the surface of this highly evolving and important field for modern society [5]. In particular, the search for greener separation methods for RE elements and actinoids, as well as the improvement of the existing ones, have recently driven the development of the separation chemistry of RE metals and actinoids [19,21,23]. However, there is still progress to be made, and based on the recent results obtained for α-amino-functionalized organophosphorus-based extractant and precipitation agents, it is highly likely that these compounds play an important role in this progress. Illustrative examples are the development of the patented Cextrant 230 functioning as an efficient and selective extractant for Ce(iv) and Th(iv) over other RE metal ions in the solvent extraction [38,39], as well as the selective separation of U(vi) and Th(iv) from RE mixtures by precipitation using only water as the solvent in acidic conditions [9]. Although the selective precipitation of RE metals from the aqueous phase has not yet been achieved with α-amino-functionalized organophosphorus compounds, it has been shown with other compounds that light and heavy REs, such as Nd and Dy, can be selectively separated by precipitation from the aqueous phase [85,86,87,88,89]. If one also considers the development of sorption materials based on the α-amino-functionalized organophosphorus compounds, as well as the tunability of their solubility, coordination affinity, and steric effects, there will certainly be many new and exciting avenues to be taken with the α-amino-functionalized organophosphorus extractants, separation agents, and sorption materials. We believe that the molecular-level knowledge obtained from the studied systems is the main driving force in this progress because, after all, it is the molecular structure of the compound that dictates its coordination affinity towards metal ions.

## Figures and Tables

**Table 1:** Synthesis strategies, separation methods, and studied metals for the α-aminophosphonate, -phosphinate, and -phosphine oxide extractants.

Extractant	Synthesis Strategy	Separation Method	Studied REs and Actinoids	Ref.
**1** MOABP	Pudovik	Solvent extraction	Y(iii), La(iii), Ce(iii), Eu(iii), Pr(iii), Tb(iii), Th(iv), U(iv), U(vi)	[31,32,33,52,53,54,55]
**2** MOCABP	Pudovik	Solvent extraction	La(iii), Ce(iii), Eu(iii), Pr(iii)	[30,31,32,33]
**3–5**	Mannich ^a^	Solvent extraction	La(iii), Lu(iii)	[34,35]
**6**	Mannich ^a^	Solvent extraction	La(iii)	[34]
**7**	Pudovik	Solvent extraction	La(iii)	[35]
**8**	Kabachnik–Fields	Solvent extraction	Sc(iii)	[11]
**9** DEHAMP	Kabachnik–Fields	Solvent extraction	Sc(iii), La(iii), Ce(iv), Gd(iii), Yb(iii), Th(iv)	[40]
**10** HEHHAP	Kabachnik–Fields	Solvent extraction, synergistic solvent extraction with Cyanex272	La(iii), Ce(iii), Pr(iii), Nd(iii), Sm(iii), Eu(iii), Gd(iii), Tb(iii), Dy(iii), Ho(iii), Y(iii), Er(iii), Tm(iii), Yb(iii), Lu(iii)	[44,48]
**11** Cextrant 230	Kabachnik–Fields	Solvent extraction	Sc(iii), La(iii), Ce(iv), Gd(iii), Yb(iii), Th(iv), U(vi)	[39,45,56]
**12** DEHAPP	Kabachnik–Fields	Solvent extraction	Sc(III), La(III), Ce(III), Ce(iv), Gd(III), Y(III), Yb(III), Th(iv)	[41]
**13** HEHAPP	Kabachnik–Fields	Solvent extraction, synergistic solvent extraction with D2EHPA	La(iii), Ce(iii), Pr(iii), Nd(iii), Sm(iii), Eu(iii), Gd(iii), Tb(iii), Dy(iii), Ho(iii), Y(iii), Er(iii), Tm(iii), Yb(iii), Lu(iii)	[42,46]
**14** HEHAMP	Kabachnik–Fields	Solvent extraction, synergistic solvent extraction with HEHEHP	Sc(iii), La(iii), Pr(iii), Nd(iii), Sm(iii), Eu(iii), Gd(iii), Tb(iii), Dy(iii), Ho(iii), Y(iii), Er(iii), Tm(iii), Yb(iii), Lu(iii)	[43,47]
**15**–**20**	Kabachnik–Fields	Precipitation	Sc(iii), La(iii), Ce(iii), Pr(iii), Nd(iii), Sm(iii), Eu(iii), Gd(iii), Tb(iii), Dy(iii), Ho(iii), Y(iii), Er(iii), Tm(iii), Yb(iii), Lu(iii), Th(iv), U(vi)	[9]
**21** EEAMPA	Kabachnik–Fields	Solvent extraction	La(iii), Ce(iii), Pr(iii), Nd(iii), Sm(iii), Eu(iii), Gd(iii), Tb(iii), Dy(iii), Ho(iii), Y(iii), Er(iii), Tm(iii), Yb(iii), Lu(iii)	[50]
**22**	Kabachnik–Fields	Solvent extraction	Sc(iii), Nd(iii), Sm(iii), Dy(iii), Yb(iii), Lu(iii)	[11,36]
**23**–**28**	Kabachnik–Fields	Solvent extraction	Sc(iii)	[11]
**29**, **31**	Kabachnik–Fields	Solvent extraction	Lu(iii)	[36]
**30**	Kabachnik–Fields	Solvent extraction	Sc(iii), La(iii), Ce(iii), Nd(iii), Sm(iii), Gd(iii), Y(iii), Lu(iii), U(vi)	[37]
**32** DEHAPO	Kabachnik–Fields	Solvent extraction	La(iii), Ce(iv), Gd(iii), Yb(iii), Th(iv)	[51]

^a^ The aminophosphonate moiety was synthesized with Kabachnik–Fields reaction.

**Table 2:** α-Amino-functionalized organophosphorus extractant systems and their metal complexes characterized by IR spectroscopy. Hyphen (-) denotes that no value was reported, slash (/) that the absorption band disappears, and n/a that the ligand lacks the functional group. Vibration modes are separated into stretching (*ν*) and bending (*δ*) as assigned in the original article, and the superscripts of the former denote whether the vibration is symmetric (s) or asymmetric (as).

Extractant	Metal	P-OH (cm^−1^)		*ν*P=O (cm^−1^)		P-O-C (cm^−1^)		N-H (cm^−1^)		Ref.
		Ligand	Complex	Ligand	Complex	Ligand	Complex	Ligand	Complex	
**1**	Ce(iii) Pr(iii)	1050–1000	weak	1208	1225–1155	1050–1000	weak	*ν*3330	*ν*3330	[30,33]
**2**	Ce(iii) Pr(iii)	1050–1000	weak	1240	1215–1160	1050–1000	weak	*ν*3300	*ν*3300	[30,33]
**9**	Ce(iv) Th(iv)	n/a	n/a	1250	1244_Ce_	*ν*^as^1014	*ν*^as^1014	-	-	[40]
					1247_Th_					
**10**	Yb(iii) Lu(iii)	*ν*^as^2438	/	1216	1206_Yb_	*ν*^as^1040 *ν*^s^973	*ν*^as^1040 *ν*^s^975	-	-	[44]
					1207_Lu_					
**10** + Cyanex272	Yb(iii)	*ν*^as^2319	/	1200	1203	*ν*1041 *ν*957	*ν*1041 *ν*954	*ν*3373 *δ*1624	*ν*3381 *δ*1615	[48]
**11**	Ce(iv) Th(iv)	n/a	n/a	1250	1200_Ce_	*ν*^as^1014	*ν*^as^1014	*ν*3451	*ν*3451	[39]
					1238_Th_					
	Sc(iii)	n/a	n/a	1230	1250	*ν*^as^1046 *ν*^s^1014	*ν*^as^1046 *ν*^s^1014	*δ*1650	*δ*1612	[45]
	U(vi)	n/a	n/a	1235	1256	*ν*^as^1016	*ν*^as^1016	*ν*3446	*ν*3446	[56]
**12**	Ce(iv)	n/a	n/a	1239	1126	*ν*^as^1043 *ν*^s^1010	*ν*^as^1043 *ν*^s^1010	*δ*1650	*δ*1600	[41]
**13**	Yb(iii) Lu(iii)	*ν*^as^2398 1643	/	1225	1175	*ν*^as^1050 *ν*^s^998	*ν*^as^1050 *ν*^s^998	*ν*3300 ^a^	*ν*3300 ^a^	[42,46]
**13** + D2EHPA	Lu(iii)	*ν*2402 1643	/	1231	1176	*ν*1031	*ν*1031	-	-	[46]
**14**	Yb(iii)	*ν*2314 *δ*981	/	1159	1204	*ν*1026	*ν*1041	-	-	[43]
**14** + HEHEHP	Yb(iii)	*ν*2317 *δ*981	/	1206	1145	*ν*^as^1039	*ν*^as^1041	*δ*1620	*δ*1615	[47]
**21**	Lu(iii)	*ν*^as^2318	/	1146	1162	n/a	n/a	*δ*1614	*δ*1644	[50]
**32**	Ce(iv)	n/a	n/a	1054	1040	n/a	n/a	3311 1675	3396, 1666, 1614	[51]

^a^ Assigned as OH vibrations in the original article.

**Table 3:** Reported chemical compositions of RE complexes of α-aminophosphonate, -phosphinate, and -phosphine oxide extractants and precipitation agents in solution. For compounds with acidic protons, HL and L denote the protonated and deprotonated versions of compounds, respectively.

	Complex	Acid	Diluent	Ref.
**1**	U(vi)O_2_L_2_ U(iv)L_4_	H_2_SO_4_	Ligroin Recryst. from ethanol	[52]
**1**	ML_3_∙HL/ML_2_∙HL_2_ (M=Eu, Tb)	HCl, HNO_3_, and HClO_4_	Ligroin	[53]
**1**	ML_3_HL (M=Ln, Eu)	HCl, HClO_4_	Petroleum ether, CHCl_3,_ CCl_4_	[31]
**1**	Ce(iii)L_3_∙2HL	HCl	CHCl_3_, benzene	[33]
**1**	Ce(iii)L_3_∙HL	HCl	CCl_4_, cyclohexane	[33]
**1**	PrL_3_∙HL	HCl	CHCl_3_, benzene, CCl_4_	[33]
**1**	PrL_3_	HCl	cyclohexane	[33]
**2**	ML_3_∙HL (M=Ln, Eu)	HCl	CHCl_3_	[31]
**2**	Ce(iii)L_3_∙2HL	HCl	CHCl_3_	[33]
**2**	PrL_3_	HCl	CHCl_3_	[33]
**3**	LaLX_3_	-	CHCl_3_	[35]
**3**	LuL_2_X_3_ or LuLX_3_ *	-	CHCl_3_	[35]
**4**	LaL_2_X_3_ or LaLX_3_ *	-	CHCl_3_	[35]
**4**	LuL_2_X_3_ or LuLX_3_ *	-	CHCl_3_	[35]
**5**	LaL_2_X_3_ or LaLX_3_ *	-	CHCl_3_	[35]
**5**	LuL_2_X_3_	-	CHCl_3_	[35]
**6**	LaL_2_Pic_3_	-	CHCl_3_	[34]
**7**	LaLX_3_	-	CHCl_3_	[35]
**9**	Ce(iv)(SO_4_)_2_ ∙ 2L	H_2_SO_4_	heptane	[40]
**9**	Th(HSO_4_)_2_SO_4_ ∙ L	H_2_SO_4_	heptane	[40]
**10**	MClH_2_L_4_ (M=Lu, Yb)	HCl	heptane	[44]
**10** + Cyanex272	MH_2_Cl_2_A_2_B (A=**10**, M=Yb, Lu)	HCl	heptane	[48]
**11**	Ce(iv)(HSO_4_)_2_SO_4_ ∙ 2L	H_2_SO_4_	heptane	[39]
**11**	Th(HSO_4_)_2_SO_4_ ∙ L	H_2_SO_4_	heptane	[39]
**11**	Sc(HSO_4_)SO_4_ ∙ 2L	H_2_SO_4_	heptane	[45]
**11**	UO_2_SO_4_ ∙ 2L	H_2_SO_4_	heptane	[56]
**12**	Ce(iv)(HSO_4_)_2_SO_4_ ∙ 2L	H_2_SO_4_	heptane	[41]
**13**	ML_3_ (M=La, Gd, Y, Lu)	HCl	heptane	[42]
**13** + D2EHPA	LuCl_2_H_4_A_3_B_2_ (A=**13**)	HCl	heptane	[46]
**14**	MH_2_ClL_4_ (M=Tm, Yb, Lu)	HCl	heptane	[43]
**14** + HEHEHP	MA_2_B_4_ (A=**14**, M=Lu, Yb, Tm, Er, Y, Ho)	HCl	heptane	[47]
**15**	LuL(NO_3_)_2_	HNO_3_	water	[9]
**15**	LaL_2_(NO_3_)	HNO_3_	water	[9]
**15**	YL_3_	HNO_3_	water	[9]
**21**	MHL_3_NO_3_ (M=La, Nd, Gd, Lu)	HNO_3_	heptane	[50]
**22**	ScL_2_X_3_	HClO_4_	toluene	[11]
**32**	Ce(iv)(HSO_4_)_2_SO_4_ ∙ L	H_2_SO_4_	heptane	[51]

* Compositions for LnX_3_ with the two different NaPic ratios: 1:250 for former and 10:1 for latter.

**Table 4:** Highest reported *SF* for the extraction of actinoids Th(iv) and U(vi) with α-amino-functionalized organophosphorus compounds. FP indicates full precipitation of the metal marked in parentheses, because of which the *SF* could not be determined.

Extractant	Ce(iv)/Th	U/Eu	Th/RE	U/RE	Th/Lu	U/Th	U/Lu	Ref.
**1**	-	26,000 *	-	-	-	-	-	[53] ^a^
**11**	14.7	-	-	-	-	-	-	[39] ^b^
**11**	-	-	>1000	>1000	-	-	-	[56] ^c^
**12**	754.2	-	-	-	-	-	-	[41] ^d^
**15**	-	-	-	-	4.50 ^g^	FP(U) ^i^	FP(U) ^i^	[9]
**16**	-	-	-	-	6.02 ^f,^^	2.01 ^e^	4.03 ^f,^^	[9]
**17**	-	-	-	-	9.17 ^g^	2.40 ^e^	8.68 ^g^	[9]
**18**	-	-	-	-	44.41 ^g^	FP(U) ^h^	FP(U) ^h^	[9]
**19**	-	-	-	-	FP(Lu) ^j^	FP(U) ^h^	FP(U) ^h^	[9]
**20**	-	-	-	-	FP(Th) ^j^	FP(U) ^e^	FP(U) ^e^	[9]
**32**	100.3	-	-	-	-	-	-	[51] ^k^

* Separation factor for U/Tb mentioned to be similar; ^ best *SF* with error smaller than the value; ^a^ 5 mM extractant **1**, 0.1 mM Eu(iii), 4 mM U(vi)O_2_^2+^, 1 M H_2_SO_4_; ^b^ 0.1 M extractant **11**, 0.01 M M(iv), 3 M H_2_SO_4_; ^c^ 0.1 M extractant **11**, 0.01 M metals, 3.23 M H^+^ for Th/RE, 0.22 M or 3.23 M H^+^ for U/RE; ^d^ 0.048 M extractant **12**, 6 mM Th, 5 mM Ce, 0.2134 M H_2_SO_4_; ^e^ 2.5 g/L extractant, 9 mg/L, pH 1; ^f^ 2.5 g/L extractant, 9 mg/L, pH 2; ^g^ 2.5 g/L extractant, 9 mg/L, pH 2.5; ^h^ 2.5 g/L extractant, 9 mg/L, pH 3; ^i^ 2.5 g/L extractant, 9 mg/L, pH 3.5; ^j^ 2.5 g/L extractant, 9 mg/L, pH 2.5; ^k^ 0.1 M extractant **32**, 0.01 M M(iv), 0.9353 M H_2_SO_4._

**Table 5:** Loading capacities of the α-amino-functionalized organophosphorus compounds used in RE and actinoid extraction studies, as reported in the original papers.

Extractant	Dilution	Metal	Acid	Capacity	Ref.
**9**	0.63 M in heptane	0.23 M Ce(iv) (∑Ce 0.24 M) 0.02 M Th(iv)	H_2_SO_4_	30.0 g/L Ce(iv) 24.4 g/L Th(iv)	[40]
**10**	30% (*v*/*v*) in heptane	0.0985 M YbCl_3_ 0.0986 M LuCl_3_	HCl	12.76 g/L Yb 15.43 g/L Lu	[44]
**11**	30% (*v*/*v*) in heptane	Ce(iv) & Th(iv)	H_2_SO_4_	>30 g/L Ce(iv) ~43 g/L Th(iv)	[39,83]
**11**	30% (*v*/*v*) in heptane	0.064 M Sc	H_2_SO_4_	3.85 g/L Sc	[45]
**11**	5% (*v*/*v*) in heptane	8.08 mM Th(iv) 21 mM U(vi)	H_2_SO_4_	4.08 g/L Th(iv) 6.16 g/L U(vi)	[56]
**12**	30% (*v*/*v*) in heptane	0.29 M Ce(iv)	H_2_SO_4_	31.43 g/L CeO_2_	[41]
**13**	30% (*v*/*v*) in heptane	0.1 M RE	HCl	0.201 M Ho 0.205 M Er 0.216 M Yb 0.229 M Lu	[42]
**14**	30% (*v*/*v*) in heptane	0.055 M YbCl_3_ *	HCl	15.17 g/L Lu 14.46 g/L Yb 12.64 g/L Y	[43]
**14** + HEHEHP	30% (*v*/*v*) in heptane (1:1 extractant ratio)	96 mM Lu 92 mM Yb	HCl	27.25 g/L Lu_2_O_3_ 26.59 g/L Yb_2_O_3_	[47]
**21**	4 mM in heptane	0.4 mM RE	HNO_3_	0.393 mM Ho 0.402 mM Er 0.422 mM Tm 0.435 mM Yb 0.450 mM Lu	[50]
**32**	30% (*v*/*v*) in heptane	0.143 M Ce(iv)	H_2_SO_4_	16.66 g/L CeO_2_	[51]

* Used RE concentration only reported for Yb.

**Table 6:** Best reported *SF* for adjacent RE elements (excluding the radioactive promethium). Precipitation studies carried out for **15**–**20** were done in water, whereas heptane was used as a diluent in all solvent extraction experiments. The detailed experimental conditions are given below.

Extractant	Ce/La	Pr/Ce	Nd/Pr	Sm/Nd	Eu/Sm	Gd/Eu	Tb/Gd	Dy/Tb	Ho/Dy	Er/Ho	Tm/Er	Yb/Tm	Lu/Yb	Y/Ho	Er/Y	Ref.
**10**	1.47	1.23	0.85	1.93	1.14	0.62	1.76	1.39	1.39	2.28	4.29	1.59	1.63	1.04	2.18	[44] ^a^
**10**	-	-	-	-	-	-	-	-	1.27	1.23	2.36	3.18	1.59	1.41	0.88	[48] ^b^
**10** + Cyanex272	-	-	-	-	-	-	-	-	2.57	3.33	3.07	3.58	1.60	1.60	2.08	[48] ^c^
**12**	135.1 *	-	-	-	-	-	-	-	-	-	-	-	-	-	-	[41] ^d^
**13**	-	-	-	-	-	-	-	-	-	2.83	3.87	5.64	4.89	2.24	2.35	[42] ^e^
**13** + D2EHPA	0.72	1.31	0.93	0.92	1.03	0.97	1.01	1.13	1.03	1.45	2.58	2.77	1.77	1.35	0.93 **	[46] ^f^
**14**	-	-	1.43	1.35	1.11	1.19	1.44	1.07	1.32	1.78	1.93	1.36	1.24	1.13	1.58	[43] ^g^
**14** + HEHEHP	-	-	1.20	1.14	1.45	1.13	1.16	1.17	1.05	2.11	1.78	1.76	1.20	1.32	1.61	[47] ^h^
**15**	2.56 ^m^	2.06 ^l^	1.16 ^l^	1.41 ^m^	1.28 ^m^	1.22 ^m^	1.01 ^n^	2.67 ^l^	1.28 ^m,^^	2.00 ^l,^^	-	2.88 ^l^	2.52 ^k^	-	3.02 ^l^	[9]
**16**	1.33 ^m,^^	1.23 ^i^	1.48 ^j^	1.50 ^k^	1.45 ^j,^^	1.52 ^j,^^	1.05 ^i^	1.23 ^m^	1.41 ^m^	1.09 ^i^	-	1.77 ^m,^^	1.30 ^n^	-	2.75 ^i^	[9]
**17**	2.92 ^j^	1.36 ^k^	1.75 ^j^	1.44 ^l^	1.76 ^l^	1.41 ^l^	0.84 ^l^	1.51 ^l^	1.44 ^l^	1.18 ^l^	-	2.22 ^l,^^	1.18 ^l^	-	3.33 ^m^	[9]
**18**	3.81 ^l^	1.26 ^m^	1.11 ^n^	1.70 ^l^	1.07 ^n,^^	1.21 ^m^	1.49 ^l^	1.50 ^l^	1.47 ^l^	1.53 ^l^	-	3.60 ^k^	2.32 ^n^	-	2.21 ^l^	[9]
**19**	2.11 ^l^	2.18 ^k^	1.54 ^k^	2.04 ^k^	1.20 ^n^	1.42 ^k^	1.14 ^k^	1.06 ^k^	1.12 ^k^	1.57 ^n^	-	4.33 ^n^	FP(Lu) ^n^	-	1.87 ^n^	[9]
**20**	1.88 ^m^	1.50 ^n^	1.15 ^m^	1.73 ^m^	1.29 ^l^	1.16 ^l^	1.77 ^l^	1.20 ^l^	1.08 ^l^	1.36 ^l^	-	1.94 ^l^	2.33 ^n^	-	2.03 ^l^	[9]
**21**	1.54	2.57	1.09	1.43	1.62	0.92	1.83	1.56	1.35	1.71	1.97	2.37	1.63	1.68	1.00	[50] ^o^
**32**	167.0 *	-	-	-	-	-	-	-	-	-	-	-	-	-	-	[51] ^p^
D2EHPA	2.14	1.07	1.06	4.86	2.23	1.69	1.60	1.42	1.24	1.70	1.50	1.30	1.03	-	-	[84] ^q^
HEHEHP	1.30	1.09	1.17	2.00	1.96	1.46	2.35	1.62	2.58	1.25	1.33	1.12	1.13	-	-	[84] ^q^

* Ce(iv) was used instead of Ce(iii); ** value for the reverse pairing reported; ^ best *SF* with error smaller than the value; ^a^ 0.1 M extractant **10**, 2 mM RE, pH 4.5; ^b^ 0.1 M extractant **10**, 1 mM RE, pH 2.5; ^c^ 0.05 M extractant **10** and 0.05 M Cyanex272, 1 mM RE, pH 2.5; ^d^ 0.048 M extractant **12**, 5.1 mM La, 5 mM Ce, 0.2134 M H_2_SO_4_; ^e^ 0.05 M extractant **13**, 5 mM RE, c(HCl): Tm/Er 2 M, Yb/Tm 4.1 M, Lu/Yb 3.7 M, Y/Ho 1 M, Er/Y 2.5 M, Er/Ho 2 M; ^f^ 0.05 M extractant (total, 1:1 molar ratio), 1 mM RE, 2.5 M H^+^; ^g^ 0.1 M extractant **14**, 5 mM RE, pH 1.0; ^h^ 0.1 M extractant (total, χ = 0.5), 0.01 M RE, pH: Y/Ho 1.3, all others 1.0; ^i^ 2.5g/L extractant, 9 mg/L RE, pH 1; ^j^ 2.5g/L extractant, 9 mg/L RE, pH 2; ^k^ 2.5g/L extractant, 9 mg/L RE, pH 2.5; ^l^ 2.5g/L extractant, 9 mg/L RE, pH 3; ^m^ 2.5g/L extractant, 9 mg/L RE, pH 3.5; ^n^ 2.5g/L extractant, 9 mg/L RE, pH 4; ^o^ 4 mM extractant **21**, 0.2 mM RE, pH 1.0; ^p^ 0.1 M extractant **32**, 0.01 M RE, 0.4353 M H_2_SO_4_; ^q^ 0.2 M extractant in kerosene, 1 g/l RE, 0.1 M HCl.

**Table 7:** The highest values of synergistic enhancement factors *R* reported for each system (and RE). The molar fraction *χ* of the α-aminophosphonate is included in parentheses.

Extractant	Ho	Er	Tm	Yb	Lu	Y	Ref.
**13** + D2EHPA	-	-	-	-	3.96 (0.5)	-	[46] ^a^
**14** + HEHEHP	2.18 (0.4)	2.14 (0.5)	2.54 (0.5)	2.76 (0.5)	2.89 (0.5)	2.14 (0.5)	[47] ^b^
**10** + Cyanex272	1.95 (0.4)	2.71 (0.6)	2.43 (0.6)	3.67 (0.5)	3.39 (0.5)	-	[48] ^c^

^a^ 0.01 M Lu(iii), 0.03 M extractant (sum), 0.6 M H^+^; ^b^ 0.02 M RE, 0.1 M extractant (sum), pH 2; ^c^ 3 mM RE, 0.03 M extractant (sum), pH 2.5.

## Data Availability

Not applicable.
